# Development and application of a risk nomogram for the prediction of risk of carbapenem-resistant *Acinetobacter baumannii* infections in neuro-intensive care unit: a mixed method study

**DOI:** 10.1186/s13756-024-01420-6

**Published:** 2024-06-13

**Authors:** Yuping Li, Xianru Gao, Haiqing Diao, Tian Shi, Jingyue Zhang, Yuting Liu, Qingping Zeng, JiaLi Ding, Juan Chen, Kai Yang, Qiang Ma, Xiaoguang Liu, Hailong Yu, Guangyu Lu

**Affiliations:** 1 Department of Neurosurgery, Neuro-Intensive Care Unit, Clinical Medical College, Yangzhou, 225001 China; 2 Neuro-Intensive Care Unit, Department of Neurosurgery, Northern Jiangsu People’s Hospital Affiliated to Yangzhou University, 225001 China; 3 School of Nursing, Yangzhou University, Yangzhou, 225009 China; 4 School of Artificial Intelligence, School of Information Engineering, Yangzhou, 225009 China; 5 Department of Neurology, Northern Jiangsu People’s Hospital, Yangzhou, 225001 China; 6 School of Public Health, Yangzhou University, Yangzhou, 225009 China; 7 Jiangsu Key Laboratory of Zoonosis, Yangzhou, 225009 China

**Keywords:** Carbapenem-resistant *Acinetobacterbaumannii* infections, Neuro-ICU patients, Mixed method study, Prediction model

## Abstract

**Objective:**

This study aimed to develop and apply a nomogram with good accuracy to predict the risk of CRAB infections in neuro-critically ill patients. In addition, the difficulties and expectations of application such a tool in clinical practice was investigated.

**Methods:**

A mixed methods sequential explanatory study design was utilized. We first conducted a retrospective study to identify the risk factors for the development of CRAB infections in neuro-critically ill patients; and further develop and validate a nomogram predictive model. Then, based on the developed predictive tool, medical staff in the neuro-ICU were received an in-depth interview to investigate their opinions and barriers in using the prediction tool during clinical practice. The model development and validation is carried out by R. The transcripts of the interviews were analyzed by Maxqda.

**Results:**

In our cohort, the occurrence of CRAB infections was 8.63% (47/544). Multivariate regression analysis showed that the length of neuro-ICU stay, male, diabetes, low red blood cell (RBC) count, high levels of procalcitonin (PCT), and number of antibiotics ≥ 2 were independent risk factors for CRAB infections in neuro-ICU patients. Our nomogram model demonstrated a good calibration and discrimination in both training and validation sets, with AUC values of 0.816 and 0.875. Additionally, the model demonstrated good clinical utility. The significant barriers identified in the interview include “skepticism about the accuracy of the model”, “delay in early prediction by the indicator of length of neuro-ICU stay”, and “lack of a proper protocol for clinical application”.

**Conclusions:**

We established and validated a nomogram incorporating six easily accessed indicators during clinical practice (the length of neuro-ICU stay, male, diabetes, RBC, PCT level, and the number of antibiotics used) to predict the risk of CRAB infections in neuro-ICU patients. Medical staff are generally interested in using the tool to predict the risk of CRAB, however delivering clinical prediction tools in routine clinical practice remains challenging.

**Supplementary Information:**

The online version contains supplementary material available at 10.1186/s13756-024-01420-6.

## Introduction

*Acinetobacter baumannii (A. baumannii)* is a significant pathogen responsible for nosocomial infections in healthcare settings [1]. The prevalence of *carbapenem-resistant A. baumannii* (CRAB) is on the rise globally due to *A. baumannii's* complex drug resistance and range of virulence factors, as well as the improper use of antibiotics [2, 3]. For example, the resistance rate of *A.baumannii* to carbapenems is as high as 88% in Europe and 85% in Latin America [4, 5]. A recent study reported resistance rates of *A.* *baumannii* to imipenem and meropenem in the intensive care unit (ICU) of a general hospital in Greece, reaching as high as 100% and 98.91%, respectively [6]. As bacterial resistance mechanisms are evolving and limiting available therapeutic options [7], this has resulted in CRAB infections are linked with higher mortality rates, longer stays in the ICU, and increased patient costs [8–10]. For example, among patients infected with CRAB who did not receive appropriate empiric antimicrobial therapy, the overall mortality rate was 86.1% [11].

Critically ill patients are susceptible to infection due to delayed immune responses and the use of invasive devices [12]. For instance, a recent study found that CRAB strains were prevalent in 71.4% of critically ill patients admitted to ICU [13]. Identification of risk factors for CRAB infections is crucial for the early implementation of an appropriate therapy and for improving clinical outcomes. Reported risk factors associated with CRAB infections include age, sex, malignancy, mechanical ventilation, hemodialysis, length of ICU stay, transfusion, and previous use of carbapenems and aminoglycosides [14–16]. However, the findings of studies investigating risk factors of CRAB infections are inconsistent. For example, a positive association between both sexes and CRAB infections has been reported [15, 17].

Critically ill patients constitute a diverse group, with significant heterogeneity among individuals [18]. Of them, a neuro-intensive care unit (neuro-ICU) is a specialist unit that offers medical care to critically ill patients with neurological and neurosurgical diseases [19]. Patients in the neuro-ICU often require extended treatment and are more likely to experience altered mental status, and aspiration, putting them at an increased risk of developing healthcare-associated infections [20]. Therefore, it is crucial to accurately predict the occurrence of CRAB infections early in patients in the neuro-ICU. A risk prediction model for CRAB infections for patients in the neuro-ICU is unavailable. Only a few studies have investigated the difficulties of medical staff in applying such prediction tools in clinical practice.

Therefore, we aimed to develop and validate a nomogram to predict the risk of CRAB infections in neuro-critically ill patients. Moreover, we aimed to provide information about the barriers to the use of such risk prediction tools in the clinical setting from the clinical staff side.

## Materials and methods

### Quantitative study

#### Study design

A retrospective cohort study was conducted on neuro-ICU at a hospital in Yangzhou city, Jiangsu Province, China between January to December 2019. Data from neuro-ICU patients were randomized into training and validation sets at a 7:3 ratio. A nomogram from the training test was created using independent risk factors to predict CRAB infections in the neuro-ICU.

### Ethics statement

This study was approved by the Medical Ethics Committee of the Clinical Medical College of Yangzhou University(2023ky129). Informed consent was waived because the study was conducted retrospectively and no interventions were applied. This study was conducted in accordance with the standards set by the Declaration of Helsinki. To protect the privacy of participants, all personal information was de-identified prior to analysis.

### Study population and definitions

Patients qualified for enrollment if they were aged ≥ 18 years. The following exclusion criteria were used: (1) the length of stay in the neuro-ICU was < 24 h; (2) CRAB was detected before the patient entered the neuro-ICU or within the first 48 h of admission in the neuro-ICU or 72 h after leaving the neuro-ICU. CRAB strains were defined as resistant if they showed resistance to at least one carbapenem antibiotic [21]. The diagnosis of nosocomial infection refers to the “Diagnostic Criteria for Hospital Infection (Trial)” issued by the Chinese Ministry of Health [22]. Patients with CRAB infections constituted the CRAB infections group, and those without CRAB infections constituted the non-CRAB infections group.

### Strain collection, strain identification and susceptibility testing

Eligible samples of sputum, urine, cerebrospinal fluid, blood, and catheters were collected and sent to the microbiology laboratory after the clinical nurse ensured that the samples were adequate and free of contamination. The specimens were then inoculated on culture plates and incubated at 37 °C for 48 h. The strains were identified using a French Mérieux VITEK 2 Compact fully automated microbiology analyzer. Drug sensitivity testing was performed by paper diffusion method and the isolates were characterized as sensitive, intermediary or resistant according to Clinical and Laboratory Standards Institute guidelines. All bacterial isolation, culture and identification were done in the microbiology department of our hospital.

### Selection of predictive variables

To include all potential predictive variables, we first searched extensively on published studies on risk factors for CRAB infections in patients admitted to the ICU, particularly through systematic review and meta-analysis. Based on the search, we assessed the available variables in electronic medical records in the study hospital. Subsequently, we organized a panel discussion with doctors from both ICUs and neuro-ICU experts to finally identify variables included in our analysis (Table S1).

### Data collection

Demographic and clinical data were collected from the included patients by researcher using hospital health information system. The following data, which may be related to CRAB infections based on the literature review and clinical experience, were extracted: patients’ general characteristics, including age, gender, comorbidities, and the length of stay in neuro-ICU; laboratory examination, including the levels of albumin, red blood cell (RBC) count, platelets, leucocyte count, procalcitionin (PCT) within the first 48 h in the neuro-ICU; treatment, including surgery, and number of antibiotics. The detailed definition of candidate variables are described in Supplement.

### Handling of missing data

Our approach was tailored according to the proportion of missing data to address missing values of variables. For instance, where the proportion remained below 5%, we adopted the mean filling method as a suitable solution. Conversely, when the proportion exceeded this threshold, we employed the multiple interpolation method to handle the missing data. In cases where the number of missing variable items surpassed 10% of the total variable items, we adopted the case deletion method [23].

### Statistical analysis

Categorical variables were presented as counts and percentages (%), while continuous variables were presented as either mean ± standard deviation or median and interquartile range. The independent samples t-test was used to compare two groups of parametric values, and the Mann–Whitney U test was used to compare two groups of nonparametric values. Categorical variables were compared using the chi-square test. Following the univariate analysis, we conducted multivariate logistic regression analysis to determine the odds ratio (OR) and 95% confidence interval (CI) of independent variables. The variables selected for the multivariate model were chosen based on their physiological relevance and statistical significance in the univariate analysis. A threshold P value of 0.25 was used in the selection process. All statistical tests were two-tailed and a significance level of 0.05 was used for the multivariate analysis. The above statistical tests were performed using Stata15 software.

A predictive nomogram model was formulated based on the results of the multivariate analysis of the training test, which allowed us to better predict CRAB infections. We evaluated the accuracy of the nomogram using an internal validation cohort. The predictive capacity of the nomogram was quantified by calculating the area under the receiver operating characteristic (ROC) curve (AUC). Accuracy, sensitivity, specificity, detection rate, positive predictive value (PPV), and negative predictive value (NPV) were used to evaluate the performance of the nomogram model. Evaluation of calibration accuracy through calibration plot. In the calibration plot, the Brier score provided the difference between the probabilistic predictions and the true results. It ranges from 0 to 1, with scores closer to 0 indicating better predictions. Calibration slope is preferred to assess calibration and is used to evaluate the agreement between observed and predicted values, with values closer to 1 indicating better performance [24]. The calibration-in-the-large was calculated as the intercept coefficient of the logistic regression with the linear predictor as an offset; zero was the ideal value [25]. Furthermore, we used decision curve analysis (DCA) to assess the effectiveness in a clinical environment. During the development and validation phase, all analyses were performed using R software (version 4.2.0, https://www.R-project.org).

### Qualitative study

We conducted a in-depth interview to explore the attitudes and barriers of clinical medical staff in using such prediction tools in neuro-ICU context. Participants were selected through convenience and purposive sampling. Data collection and analysis were carried out simultaneously until data saturation was reached. The interviews were transcribed verbatim within 24 h of the interview and reviewed for accuracy by the interviewer. All materials, including interviews, original transcripts, and data analysis, were written in Chinese.

The study utilized a semi-structured format with open-ended questions, which was developed by the research team based on relevant literature and an intensive group involving clinical experts and epidemiologistis. Illustrative questions were: “what is your overall opinion regarding clinical risk prediction model?”; “what are the difficulties you may encounter in using this model in your clinical work?” Probing questions, such as “please elaborate on that,” were used to elicit more information. The transcripts of the interviews were analyzed by Maxqda.

## Results

### Quantitative results

#### Basic characteristics

**Fig. 1:**
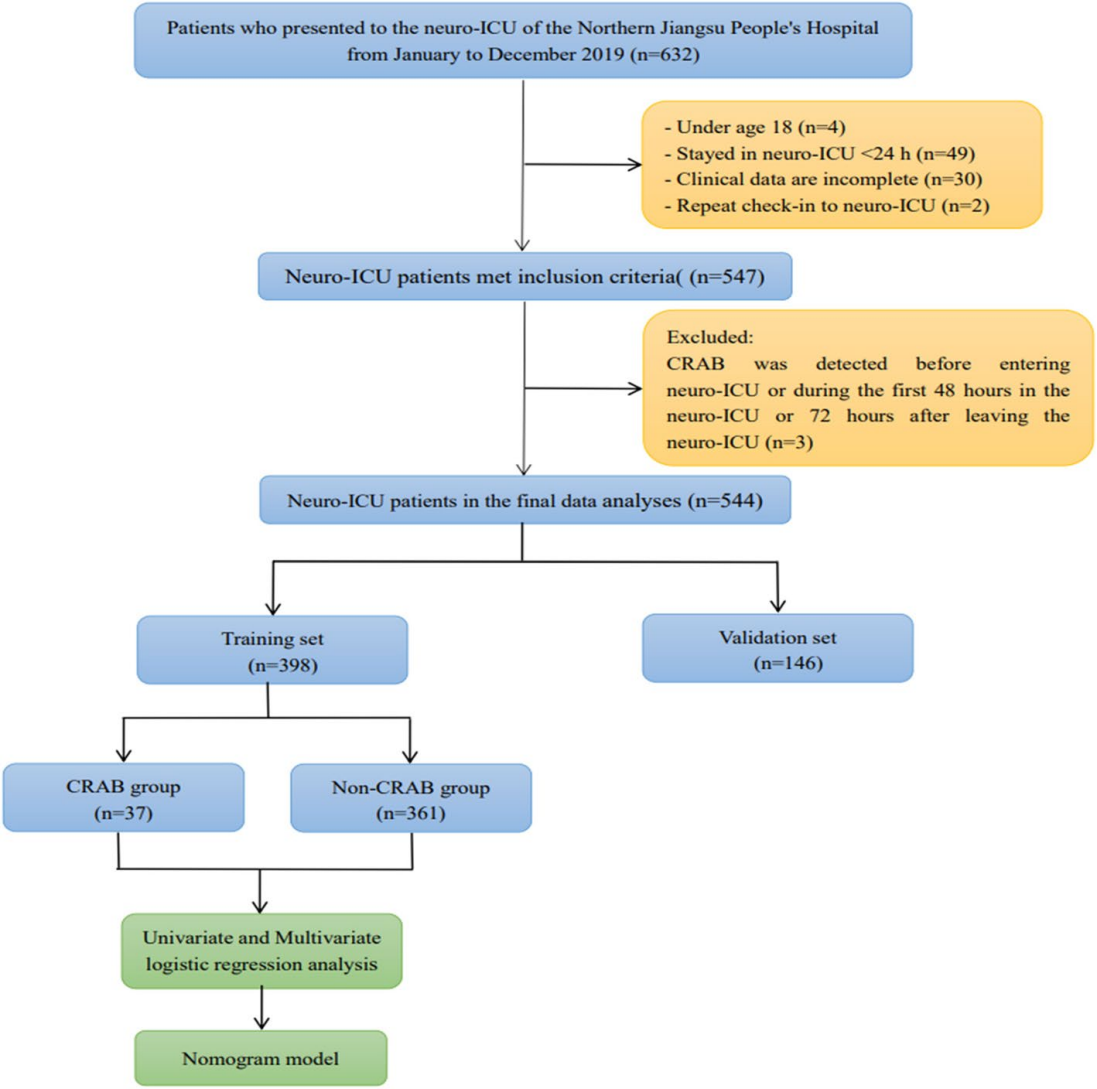
Study flow diagram

Among the 632 patients admitted to the neuro-ICU from January to December 2019, 544 patients were eventually eligible and included in the statistical analysis (Fig. 1). Table 1 provides a summary of the main demographic and clinical characteristics of the participants.

**Table 1:** Demographic and clinical characteristics of the neuro-ICU patients

**The Variables**	**Total number**	**The CRAB group**	**The Non-CRAB group**
Rate n(%)	544	47(8.6)	497(91.4)
**Clinical characteristics**			
Gender			
Female n (%)	213(39.2)	7(14.9)	206(41.4)
Male n (%)	331(60.8)	40(85.1)	291(58.5)
Age, years Mean ± SD	60.31(13.09)	60.36(11.66)	60.30(13.23)
Length of stay in neuro-ICU, days Mean ± SD	7.37(7.65)	13.62(10.58)	6.77(7.04)
Weight, kg, Mean ± SD	66.58(12.25)	67.21(10.34)	66.52(12.42)
Hypertension, yes (%)	257(47.2)	23(48.9)	234(47.0)
Diabetes, yes (%)	78(14.3)	14(29.7)	64(12.8)
Cerebral infarction, yes (%)	50(9.2)	2(4.2)	48(9.6)
Heart disease, yes (%)	27(5.0)	0(0)	27(5.4)
BMI^1^, kg/m^2^, Mean ± SD	23.96(3.78)	23.25(3.43)	23.95(3.65)
GCS^2^ score, Mean ± SD	6.91(3.67)	6.09(3.03)	6.99(3.72)
Admission season^3^, n (%)			
Spring	127(23.3)	12(25.5)	115(23.1)
Summer	141(25.9)	12(25.5)	129(25.9)
Autumn	169(31.0)	11(23.4)	158(31.7)
Winter	107(19.6)	12(25.5)	95(19.1)
**Laboratory examinations**			
Albumin level, g/L Mean ± SD	40.88(6.82)	39.10(7.20)	41.05(6.77)
RBC^4^, 10^12^/L Mean ± SD	4.14(0.84)	3.89(0.88)	4.16(0.83)
Platelets, 10^9^/L Mean ± SD	175.83(117.04)	174.30(80.23)	175.98(119.99)
Procalcitonin, ng/ml Mean ± SD	1.64(4.25)	2.84(3.98)	1.52(4.26)
WBC^5^ count, 10^9^/L Mean ± SD	13.51(8.32)	13.10(5.78)	13.55(8.53)
ALT^6^, U/L Mean ± SD	35.09(46.47)	33.34(23.86)	35.25(48.07)
AST^7^, U/L Mean ± SD	44.67(62.85)	42.57(35.35)	44.87(64.87)
Uric Acid, umol/L Mean ± SD	294.40(109.71)	295.96(132.24)	294.26(107.49)
Urea, mmol/L Mean ± SD	5.90(3.29)	7.31(3.73)	5.77(3.21)
PH value Mean ± SD	7.39(0.12)	7.42(0.07)	7.39(0.13)
Blood glucose value, mmol/L Mean ± SD	9.71(4.29)	9.57(3.60)	9.73(4.35)
INR^9^, Mean ± SD	1.17(0.79)	1.12(0.24)	1.17(0.82)
**Treatment**			
Surgery, yes (%)	320(58.8)	25(53.1)	295(59.3)
Number of types of antibiotic, ≥ 2 (%)	93(17.1)	25(53.1)	68(13.6)

The quantitative data are distributed normally, expressed by means ± standard deviations

The quantitative data were skewed and expressed in the median (25–75% percentile); Qualitative data were expressed in n%; *IQR*: interquartile range. 1: *BMI*: Body mass index; 2: *GCS*: Glasgow coma scale; 3: Admission season: spring (March to May 2019), summer (June to August 2019), autumn (September to November 2019), winter (January, February and December 2019); 4: *RBC*: Red blood cell; 5: *WBC*: White blood cell; 6: *ALT*: Alanine transaminase; 7: *AST*: Aspartate aminotransferase; 8: *INR*: International normalized ratio

### Incidence of CRAB infections

The total incidence of CRAB infections was 8.63% (47/544), with an incidence of 9.29% (37/398) in the training set and 6.84% (10/146) in the validation set. The CRAB strains were first detected from sputum in 41 of the infected patients, in urine culture in five patients, and from other sources in one patient.

### Results of univariate and multivariable analyses

Univariate analysis indicated that the male, low GCS score, long length of stay in neuro-ICU, diabetes, low ALB level, low RBC count, high PCT level, high urea value, high PH value, low blood glucose value, and the number of types of antibiotics ≥ 2 were significantly associated with CRAB infections (Table 2). Results of multivariate analysis demonstrated that the following six variables were independent predictors for CRAB infections, including the male sex (OR:3.11; 95% CI: 1.13–8.54, *P* = 0.027), long stay in neuro-ICU (OR:1.07; 95% CI:1.02–1.11, *P* = 0.001), diabetes (OR:3.05, 95% CI:1.19–7.84, *P* = 0.020), low RBC (OR:0.54; 95% CI: 0.30–0.98, *P* = 0.043), high PCT level (OR:1.07; 95% CI:1.00–1.15, *P* = 0.049), and the number of antibiotics ≥ 2 (OR: 3.92; 95%CI:1.65–9.30, *P* = 0.002).

**Table 2:** Risk factors for neuro-ICU inpatients in the training set

**Variable**	**Univariate logistic regression analysis**		**Multivariate logistic regression analysis**	
	**OR (95%*CI)***	***P *value**	**OR (95%*CI)***	***P *value**
**Gender**				
Female	Reference			
Male	3.46(1.41–8.52)	0.007	3.11(1.13–8.54)	0.027
Age, years	0.99(0.97–1.02)	0.809		
Length of stay in neuro-ICU, days	1.08(1.05–1.13)	0	1.07(1.02–1.11)	0.001
BMI, kg/m^2^	0.94(0.85–1.04)	0.263		
GCS score	0.92(0.83–1.02)	0.122	0.90(0.79–1.03)	0.156
**Hypertension**				
No	Reference			
Yes	1.19(0.60–2.34)	0.614		
**Diabetes**				
No	Reference		Reference	
Yes	2.30(1.05–5.04)	0.037	3.05(1.19–7.84)	0.020
**Heart disease**				
No	Reference			
Yes	1			
**Cerebral infarction**				
No	Reference			
Yes	1			
**Admission season**				
Spring	Reference			
Summer	0.90(0.34–2.38)	0.840		
Autumn	0.83(0.32–2.35)	0.714		
Winter	1.30(0.49–3.46)	0.596		
Albumin level, g/L	0.97(0.93–1.01)	0.186	1.05(0.97–1.14)	0.203
RBC count, 10^12^/L	0.68(0.48–0.99)	0.045	0.54(0.30–0.98)	0.043
Platelets 10^9^/L	0.99(0.99–1.002)	0.832		
WBC count 10^9^/L	1.002(0.96–1.03)	0.906		
Procalcitonin, ng/ml	1.05(0.99–1.11)	0.063	1.07(1.00–1.15)	0.049
ALT, U/L	0.99(0.98–1.007)	0.691		
AST, U/L	0.99(0.99–1.004)	0.951		
Uric Acid, umol/L	1(0.99–1.004)	0.550		
Urea, mmol/L	1.08(1.001–1.17)	0.047	1.04(0.96–1.13)	0.277
PH value	96.65(0.81–11,487.2)	0.061	74.56(0.41–13,315.61)	0.103
Blood glucose value, mmol/L	0.94(0.85–1.03)	0.211	0.89(0.79–1.01)	0.073
INR	0.88(0.49–1.60)	0.696		
**Surgery**				
No	References			
Yes	0.90(0.45–1.78)	0.766		
**Number of types of antibiotic**				
< 2	References			
≥ 2	6.00(2.96–12.16)	0	3.92(1.65–9.30)	0.002

### Development of the nomogram

**Fig. 2:**
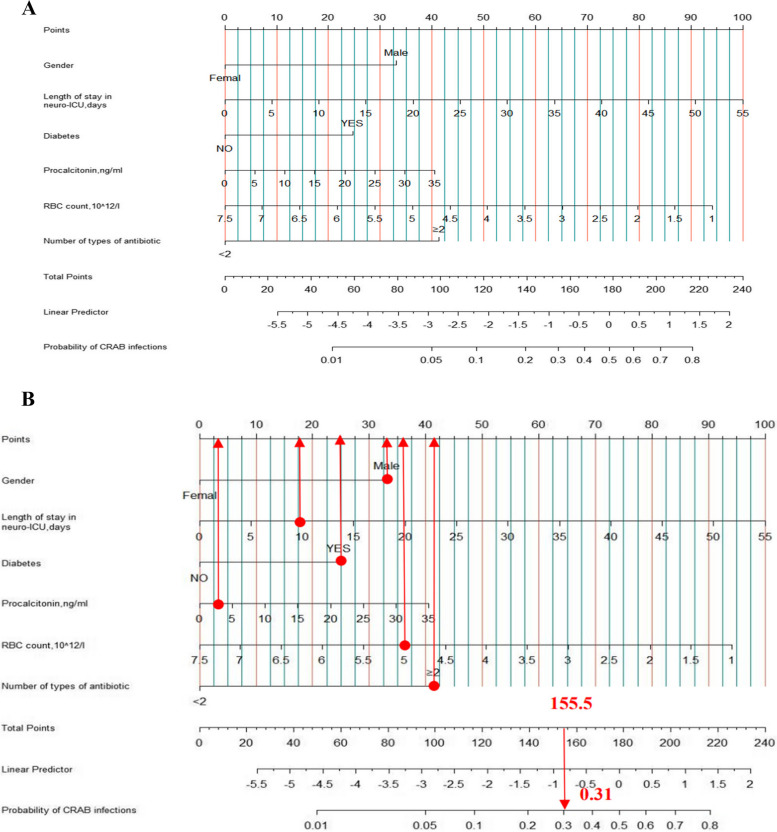
**A** Nomogram to predict the risk of CRKP infection in neuro-ICU patients. **B** Example of an application of the nomogram to predict the occurrence of CRKP infection in neuro-ICU patients

We developed a nomogram, as shown in Fig. 2A using independent risk factors to predict the risk of CRAB infections in neuro-ICU patients. To demonstrate the use of the nomogram, consider a male patient who was admitted for 10 days, was on two or more antibiotics, had diabetes, and his PCT measurement was 2.5 ng/ml with an RBC count of 5 × 10^12^/L. Using the legend, the total score for this patient was 155.5, which corresponded to a CRAB infection risk of approximately 31%.

### Validation of the nomogram

**Fig. 3:**
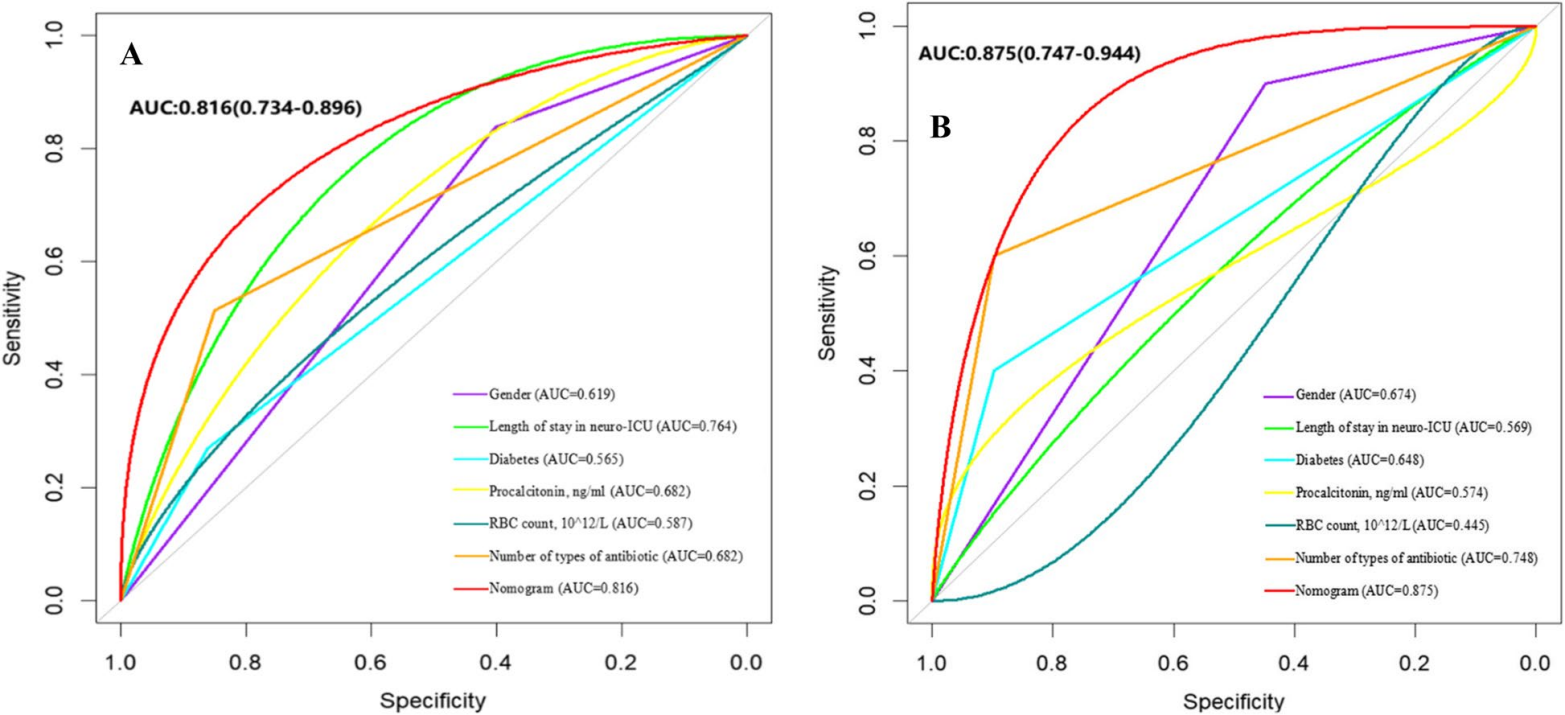
**A** The ROC curves of the nomogram model in the training set. **B** The ROC curves of the nomogram model in the validation set

**Fig. 4:**
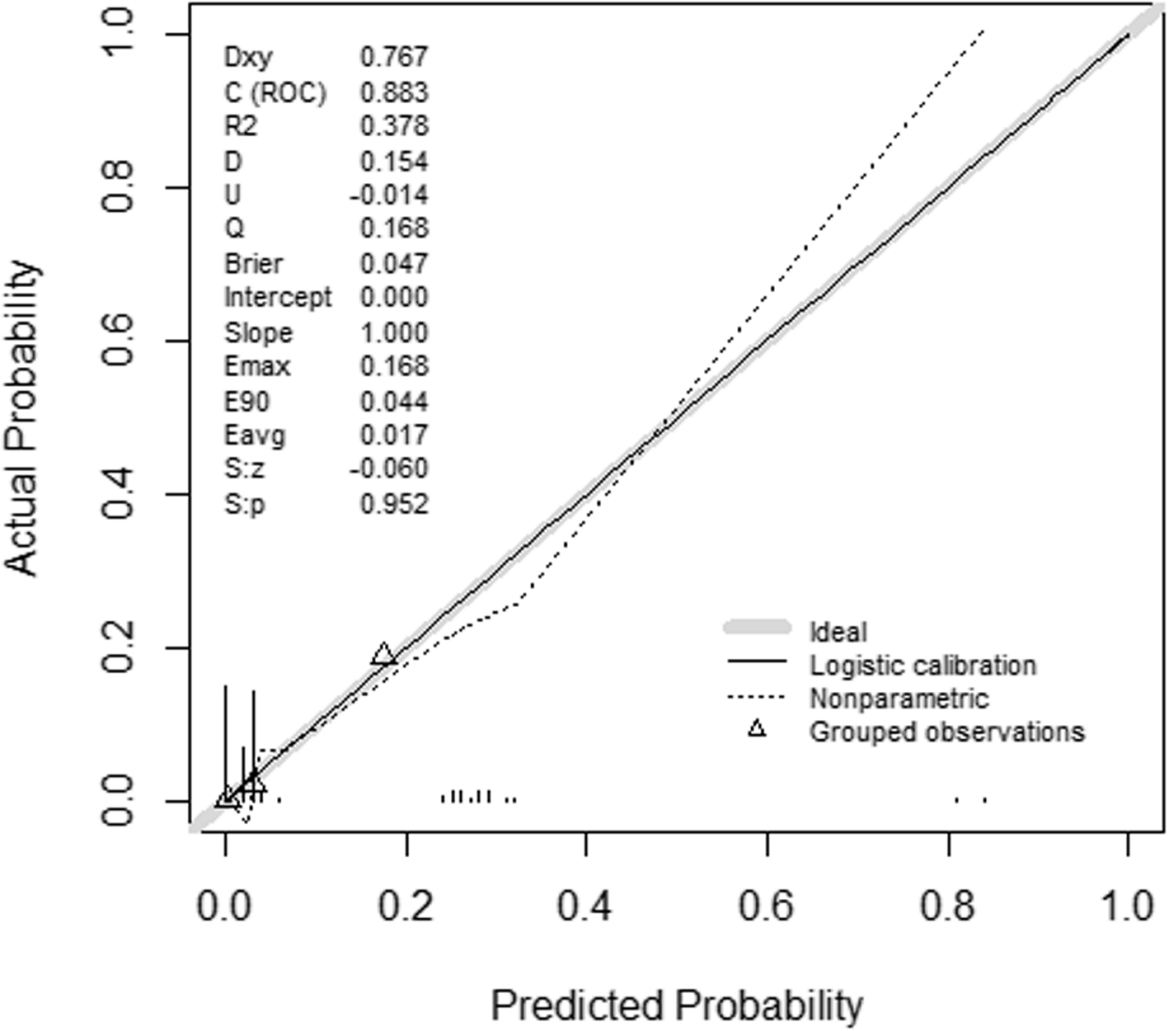
The calibration curve of the nomogram model in the validation set

In the training cohort, the AUC achieved using the nomogram model was 0.816 (95% CI: 0.734–0.896). In the validation cohort, the predictive power of the nomogram was confirmed internally. The AUC of the nomogram was 0.875 (95% CI 0.747–0.944), indicating a good discrimination. In addition, Fig. 3 shows that the AUCs of both groups indicate that the nomogram model outperforms individual risk factors in terms of AUC. Table 3 shows that the accuracy, sensitivity, detection rate, and PPV of the nomogram model for predicting CRAB infections are better, with an accuracy of 0.899, a sensitivity of 0.983, and a PPV of 0.913. These values indicate that the model can well recognize neurocritical patients who develop CRAB infections. The calibration plot showed that apparent and bias-corrected lines deviated slightly from the ideal line, indicating that the predictions were in line with the actual results. We evaluated the nomogram model. Its Brier score was 0.047; calibration slope was 1, and calibration-in-the-large was 0, indicating that the model had good prediction accuracy (Fig. 4).

**Table 3:** The predictive performance of the model in the internal validation set

Internal validation set						
	Accuracy	Sensitivity	Specificity	Detection Rate	PPV	NPV
Nomogram	0.899	0.983	0.200	0.925	0.913	0.333

*PPV* Positive Predictive Value, *NPV* Negative Predictive Value

### Evaluation of the clinical applicability of the nomogram

**Fig. 5:**
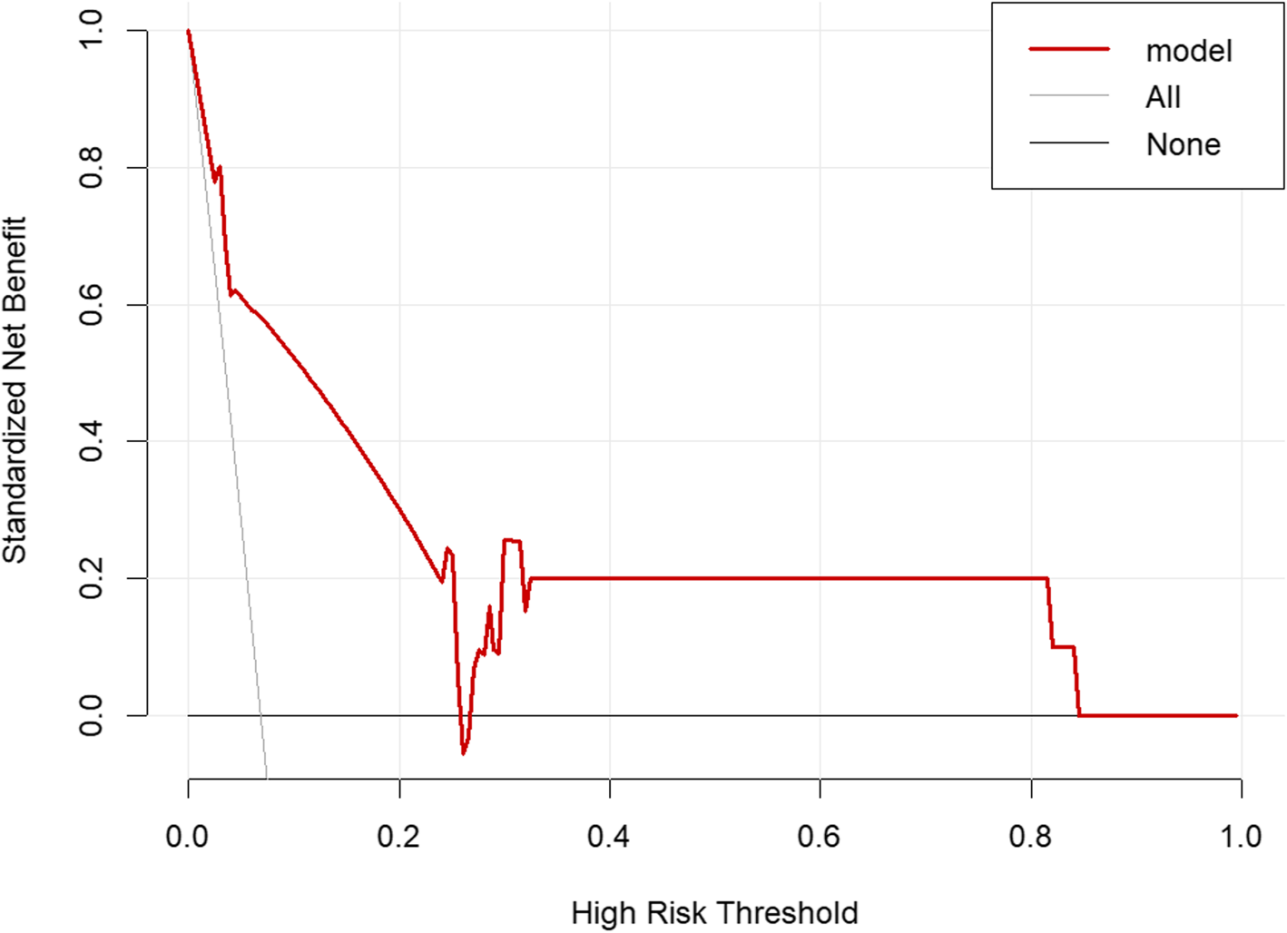
Decision curve analysis of the nomogram model

The DCA of the validation set revealed that using this predictive nomogram was more favorable for patients when the threshold probability was set between 30 and 82%. This is in contrast to the extreme approaches of detecting CRAB infections in all patients or no detection. These findings indicate that the present model offers a greater net benefit for predicting CRAB infections development across a relatively wide Fig. 5.

### Qualitative results

A total of 10 medical staff, comprising 3 nurses and 7 physicians (5 females and 5 males), aged between 26 and 60 years, participated in the qualitative interviews. Their professional tenures in neuro-ICU ranged from 2 and 19 years. Three major barriers involving in the use of nomogram models identified here include “skepticism about the accuracy of the model”, “delay in early prediction by the indicator of length of neuro-ICU stay”, and “lack of a proper protocol for clinical application”.

The medical staff mainly concerned about the accuracy of the model when considering the use of the model into clinical practice. They expected the prediction models could be fully validated before its use. Importantly, over half of the participants mentioned that “the length of stay in the neuro-ICU” although is an important predictor, it is often recorded in a rather late stage. Therefore, it may not contribute to an early prediction. Finally, the medical staff stated that currently there is no acknowledged protocol in using the prediction models in the clinical practice. For example, considering when and how to use and interpret the model, a proper guidance may needed.

## Discussion

In this study, we developed a nomogram to predict the risk of CRAB infections in neuro-critically ill patients. The model is based on easily obtainable indicators such as the length of neuro-ICU stay, male, diabetes, RBC, PCT level, and the number of antibiotics used. We validated the prediction model and found it to have good overall predictive value. It may serve as a useful tool for medical staff to predict the risk of CRAB infections among neuro-ICU patients.

Individualized predictive models can help clinical staff in the neuro-ICU to make early predictions regarding the onset of CRAB infection and implement targeted interventions. The model established had high sensitivity and low specificity, reflecting that the sensitivity and specificity were inversely proportional. The higher the sensitivity, the lower the specificity, and vice versa [26]. A higher sensitivity of the predictive model is crucial to predict CRAB infections in the neuro-ICU. Lower specificity of the predictive model may lead to some false-positive results [27], However, it can benefit critically ill patients as the screening and treatment measures usually follow in critical care contexts.

The study found that patients admitted to neuro-ICU with diabetes have an increased risk of CRAB infections. This may be due to the negative impact of diabetes on the immune system, which hinders humoral and cell-mediated immune responses, thus making it more difficult to eradicate pathogens [28]. A meta-analysis also suggested that individuals with type 2 diabetes are more likely to develop infections caused by resistant bacteria compared with those without diabetes [29]. Glucose control is a hot topic in the field of neurological care, given that neuro-critically ill patients are very sensitive to hyperglycemia [30]. Strict glycemic control in the past was considered a standard therapeutic intervention, as a large clinical trial by van den Berghe et al. in 2001 suggested that strict glucose control (< 110 mg/dL) reduces mortality in critically ill patients [31]. However, the NICE-SUGAR study published in 2009 showed that strict glucose control led to increased mortality secondary to hypoglycemia [32]. Later, a systematic review and meta-analysis of 16 clinical trials on optimal glycemic control in neurocritical care patients, revealed that strict glycemic control (70–140 mg/dL) had no effect on patient mortality but it increased the incidence of hypoglycemia [33]. Until recently, the ADA stated that a blood glucose level of 110–140 mg/dL may be appropriate if significant hypoglycemia can be avoided [34].

Our study has further highlighted that using more than two antibiotics is associated with the risk of CRAB infections. A previous study [35] suggested that three or more antibiotic classes were a risk factor for multidrug-resistant *A. baumannii* infections. This might be attributed to the fact that combination antibiotic treatment causes dysbiosis in the microbiota, whereby the majority of susceptible bacteria are eradicated, while the antibiotic-resistant strains survive [36]. Additionally, inappropriate combination antibiotic treatment can create selective pressure, thus increasing the likelihood of antibiotic-resistant infection and promoting the selection of drug-resistant bacteria [37]. Substantial debate about the differences in resistance when patients with infections are treated with combination antibiotics versus monotherapy exists. In a large retrospective study, Kosmidis et al. reported that compared to patients with nosocomial pneumonia and patients in the ICU who received combination antibiotic therapy and those treated with monotherapy were more likely to develop drug resistance [38]. However, not all studies support combination therapy to prevent drug resistance. In a prospective randomized trial of 280 adults with severe bacterial infections, Cometta et al. showed that patients treated with imipenem in combination with netimicin developed resistance more frequently than patients treated with imipenem monotherapy [39]. A subsequent meta-analysis of eight clinical trials showed that aminoglycoside/beta-lactam combination therapy had no beneficial effect on the development of antimicrobial resistance in patients with severe infections compared to beta-lactam monotherapy [40]. In general, the use of combination therapy or monotherapy should be determined based on the case, the severity of the disease, the likely pathogenic microorganisms, and local microbiome and resistance patterns.

Higher PCT levels are significant indicators of CRAB infections. PCT is a well-known marker of inflammation that rises rapidly in response to pro-inflammatory stimuli caused by bacteria, and its upregulation suggests the presence of a systemic bacterial infection [41]. Most studies affirm the value of PCT as an effective marker for early diagnosis of infection in critically ill patients [42, 43]. The results of the study further suggested that health professionals should be considered as a sign of increased susceptibility to CRAB infections when patients are in a state of a higher PCT level at the time of admission to the neuro-ICU. However, significant differences in the results of previous studies evaluating PCT for the prediction of bacterial infections exist. One study showed that significantly elevated PCT levels (1.2 ~ 3.6 ng/mL) may be a biomarker for the diagnosis of Wegener’s granulomatous bacterial infections [44], and another study showed that PCT levels > 0.22 ng/mL hint at bacterial infection in patients with vasculitis [45]. This may be related to specific patient groups and different etiologies of infection. Clinicians can perform PCT testing regularly to reduce the risk of CRAB infections.

The clinical use of the predictive model established in this study lacked external validation despite having a good performance and being well-received by medical personnel. External validation should be planned early, as it probably requires data from collaborators and datasets from other centers in different settings. For example, the prevalence of CRAB strains may vary in patients admitted to the ICU across different provinces in China, ranging from 10% in Jiangsu province to 70% in Hunan province [46]. Therefore, the model’s predictive accuracy established could be considered for further testing in these settings. Data from some large, openly, accessible databases, such as the MIMIC-IV database, could also be considered a source for external validation [47, 48].

The medical staff is mainly concerned about the application of the predictive model in their clinical practice. In the Shunde Hospital in Guangdong province, the researchers and clinical staff used an individualized artificial intelligence survival predictive system to generate individual survival curves for patients with lung adenocarcinoma [49]. This artificial intelligence predictive system could directly convey treatment benefits by comparing individual mortality risk curves under different treatment conditions. To promote the easy use of the risk predictive models, freely available, high-quality mortality risk prediction smartphone applications have been used by healthcare professionals to make evidence-based decisions in critical care environment [50]. Integrating well-performed predictive models into the system may promote the use of predictive models in primary care practice. For example, a study in New Zealand integrated a web-based decision support system with primary care electronic medical record software and found that these computerized risk prediction tools increased user visits [51].

### Strengths and limitations

Although the variables used in the constructed model are easy to measure and showed good discrimination and calibration in both the training and validation sets, some limitations should be admitted. Firstly, this is a retrospective study that may affect the stability of the CRAB infections prediction model compared to the existing predictions. Second, although the data was from one large tertiary hospital in central China, their ability to represent the general population was limited, especially considering geographical variations in CRAB strains and antibiotic resistance patterns. Therefore, future studies with multiple centers and larger populations from different regions should be conducted to improve the clinical generalizability of the model. Lastly, we only qualitatively explored the opinions and barriers to using the CRAB predictive models established in this study, and we did not conduct surveys to quantify skepticism, perceived utility, and other barriers among a broader range of medical staff, which could complement the qualitative findings and inform more targeted interventions.

## Conclusion

The study established a nomogram to predict CRAB infections in neuro-ICU patients with good accuracy. The indicators of the nomogram included the length of neuro-ICU stay, male, diabetes, RBC count, PCT level, and number of antibiotics ≥ 2. The model established here may work as an important tool for early detection and prevention of CRAB infections in neuro-critically ill patients. Medical staff are generally interested in using the tool to predict the risk of CRAB, however, delivering clinical prediction tools in routine clinical practice remains challenging.

## Supplementary Information

Supplementary Material 1.

## Data Availability

No datasets were generated or analysed during the current study.

## Abbreviations

CRAB: Carbapenem-resistant Acinetobacter baumannii
A.baumannii: Acinetobacter baumannii
ICU: Intensive care unit
Neuro-ICU: Neurointensive care unit
HAIs: Healthcare-associated infections
OR: Odds ratio
CI: Confidence interval
ROC: Receiver operating characteristic
AUC: Area under the curve
DCA: Decision curve analysis
SD: Standard deviation
IQR: Interquartile range
INR: International Normalized Ratio
GCS: Glasgow coma score
BMI: Body mass index
RBC: Red blood cell
WBC: White blood cell
ALT: Alanine transaminase
AST: Aspartate transaminase
PCT: Procalcitonin
PPV: Positive Predictive Value
NPV: Negative Predictive Value
