# Correction: Development and application of a risk nomogram for the prediction of risk of carbapenem-resistant *Acinetobacter baumannii* infections in neuro-intensive care unit: a mixed method study

**DOI:** 10.1186/s13756-024-01469-3

**Published:** 2024-09-27

**Authors:** Yuping Li, Xianru Gao, Haiqing Diao, Tian Shi, Jingyue Zhang, Yuting Liu, Qingping Zeng, JiaLi Ding, Juan Chen, Kai Yang, Qiang Ma, Xiaoguang Liu, Hailong Yu, Guangyu Lu

**Affiliations:** 1 Department of Neurosurgery, Neuro-Intensive Care Unit, Clinical Medical College, Yangzhou, 225001 China; 2 Neuro-Intensive Care Unit, Department of Neurosurgery, Northern Jiangsu People’s Hospital Affiliated to Yangzhou University, Yangzhou, 225001 China; 3 School of Nursing, Yangzhou University, Yangzhou, 225009 China; 4 School of Artificial Intelligence, School of Information Engineering, Yangzhou, 225009 China; 5 Department of Neurology, Northern Jiangsu People’s Hospital, Yangzhou, 225001 China; 6 School of Public Health, Yangzhou University, Yangzhou, 225009 China; 7 Jiangsu Key Laboratory of Zoonosis, Yangzhou, 225009 China

**Antimicrobial Resistance & Infection Control (2024) 13:62**

10.1186/s13756-024-01420-6

The original article mistakenly presented the affiliations of co-authors, Yuping Li’s and Guangyu Lu’s the wrong way around due to an error carried forward during production. The affiliations have since been corrected.

